# Culture‐acquired genetic variation in human pluripotent stem cells: Twenty years on

**DOI:** 10.1002/bies.202400062

**Published:** 2024-06-14

**Authors:** John P. Vales, Ivana Barbaric

**Affiliations:** ^1^ Centre for Stem Cell Biology School of Biosciences University of Sheffield Sheffield UK; ^2^ Neuroscience Institute University of Sheffield Sheffield UK; ^3^ INSIGNEO Institute University of Sheffield Sheffield UK

**Keywords:** genetic stability, human pluripotent stem cells, mutation, recurrent variants, selection

## Abstract

Genetic changes arising in human pluripotent stem cells (hPSC) upon culture may bestow unwanted or detrimental phenotypes to cells, thus potentially impacting on the applications of hPSCs for clinical use and basic research. In the 20 years since the first report of culture‐acquired genetic aberrations in hPSCs, a characteristic spectrum of recurrent aberrations has emerged. The preponderance of such aberrations implies that they provide a selective growth advantage to hPSCs upon expansion. However, understanding the consequences of culture‐acquired variants for specific applications in cell therapy or research has been more elusive. The rapid progress of hPSC‐based therapies to clinics is galvanizing the field to address this uncertainty and provide definitive ways both for risk assessment of variants and reducing their prevalence in culture. Here, we aim to provide a timely update on almost 20 years of research on this fascinating, but a still unresolved and concerning, phenomenon.

## Graphical abstract

Human pluripotent stem cells (hPSCs) recurrently acquire genetic changes that can impact their use in research and clinics. Here, we provide an overview of hPSC genetic variants and the mechanisms underpinning their occurrence. We highlight the need for future developments in suppressing the variant appearance and interpreting their functional implications.

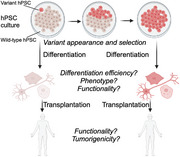

## INTRODUCTION

Regenerative therapies involving the transplantation of hPSC‐derived tissues require the propagation of billions of cells, necessitating prolonged in vitro culture to meet this demand.^[^ ^1^ ^]^ However, observationally, hPSCs that are grown over high passage numbers tend to acquire genetic changes that bestow them with altered phenotypes.^[^ ^2^ ^]^ In turn, if such genetic changes are not routinely detected and eliminated during the production of cells for therapies or bioengineered tissues, the transplantation of genetically variant cells to patients may pose a risk of reduced efficacy of therapies, and more concerningly, a safety risk due to potential tumorigenic properties of variant cells.^[^ ^3^ ^]^ Moreover, genetic changes in hPSCs could also impact basic research because the experimental manipulation of variant hPSCs possessing aberrant proliferation rates or varying differentiation abilities may generate unpredictable results, rendering stem cell‐based disease modeling, and developmental studies unreliable.^[^ ^3^ ^]^

Discarding cultures in which genetic variants are detected represents one potential solution to this issue. However, the specific applications of hPSCs, particularly in medicine, often make discarding a batch of cells impractical and inefficient, due to the high costs and timelines associated with manufacturing cell therapies. On the other hand, some of the detected changes may be inconsequential for specific uses of hPSCs, in which case the discarding of cultures may also be unwarranted and wasteful. Finally, limitations in the sensitivity of methods used for detecting genetic changes mean that variants often go undetected in culture.^[^ ^4^ ^]^ For these reasons, the safe and efficacious use of hPSCs in medicine and research necessitates a thorough understanding of the types of genetic changes arising in hPSCs, the intrinsic and extrinsic factors contributing to the appearance of genetic changes, and their consequences both for hPSCs and their differentiated progeny.

In this review, we will summarize the current knowledge of the nature and frequency of aberrations in hPSCs. Further, we will review studies focusing on the mechanistic understanding of processes that cause genetic changes in hPSCs. Finally, we will discuss potential strategies for curbing the appearance of variants in hPSCs and interpreting their functional significance for clinical use or research.

## THE SPECTRUM AND RECURRENCE OF CULTURE‐ACQUIRED VARIANTS IN HPSCS

Recurrent genetic changes in hPSCs were first reported in 2004, in a seminal study that identified culture‐acquired gains of chromosomes 12 and 17.^[^ ^5^ ^]^ At the time, it was known that mouse PSCs also acquire karyotypic aberrations,^[^ ^6^, ^7^ ^]^ particularly through amplification of chromosome 8,^[^ ^7^ ^]^ but also chromosome 11,^[^ ^7^ ^]^ which is largely syntenic to human chromosome 17. However, considering that mouse PSCs were derived 17 years before the human ones, the number of studies that reported and investigated the recurrent aberrations in mouse PSCs over that time was surprisingly low. Karyotypically abnormal mouse PSCs were recognized as a barrier for germ line transmission in gene targeting,^[^ ^6^, ^7^ ^]^ but the appearance of aberrations was nowhere near as big a concern for mouse PSC applications as for the hPSCs ones. In fact, the observation by Draper et al (2004).^[^ ^5^ ^]^ that the same karyotypic changes detected in hPSCs were characteristic of embryonal carcinoma cells, a pluripotent cell population of germ cell tumors teratocarcinomas,^[^ ^5^ ^]^ prompted concerns about the potential safety and utility of hPSCs, and highlighted the need for regular monitoring of their karyotypes during culture.

Subsequent studies probing the hPSC karyotypes documented further numerical and structural aberrations of hPSC chromosomes, but the aberrations overall converged on a defined set of abnormalities, including amplifications of regions of chromosomes 1, 12, 17, 20, and X, and losses of parts of chromosomes 10 and 18.^[^ ^8^, ^9^, ^10^ ^]^ The use of methods with finer resolution capabilities, such as CGH arrays and SNP arrays, additionally revealed smaller copy number variants (CNVs) in hPSC genomes and highlighted the gain of chromosome 20q11.21 as the most common sub‐karyotypic abnormality in hPSCs.^[^ ^10^, ^11^, ^12^ ^]^ Finally, more recently, whole genome^[^ ^13^ ^]^ and exome sequencing^[^ ^14^ ^]^ alongside analysis or RNAseq data^14^, ^15^ ^]^ were used to identify frequent single nucleotide variations (SNVs) in hPSCs. Even at the SNV level, there appears to be a common set of variants, particularly in genes such as the cancer‐related genes *Tumor Protein P53* (*TP53)* and *BCL6 corepressor* (*BCOR)*.^[^ ^13^, ^14^, ^15^ ^]^

Together, the existence of different types of genetic changes in hPSCs on the one hand implies differing mechanistic origins that may be at work, such as mitotic errors for whole chromosome gains/losses and errors in DNA replication and repair for CNVs and SNVs. On the other hand, the commonality of aberrations across the genome and across multiple samples, suggests that the resulting changes are then acted on by culture‐induced selection pressures to give rise to the recurrent aberrations.

## MUTATION: ADDRESSING HPSCS’ PROPENSITY FOR ACQUIRING GENETIC CHANGES

### Low chromosome segregation fidelity gives rise to frequent mitotic errors

The presence of whole chromosome gains and losses in abnormal hPSC karyotypes,^[^ ^16^ ^]^ implies issues with chromosome segregation during hPSC mitoses. Time‐lapse tracking of individual hPSCs as they progressed through mitosis, highlighted a relatively high incidence (∼14%−30%) of mitotic errors.^[^ ^17^, ^18^ ^]^ The high frequency of mitotic errors does not appear to be a consequence of in vitro culture of hPSCs. Recent studies suggested that as many as 80% of in vitro fertilization preimplantation embryos harbor aneuploid cells, and about 70% of these are caused by errors during mitosis, implying that high rates of mitotic errors may be an innate feature of pluripotent cells.^[^ ^19^ ^]^ In comparison to somatic cells, it has also been shown that hPSCs exhibit significantly higher rates of mitotic errors caused wholly by lagging chromosomes with improper spindle‐kinetochore attachments or “merotelic attachments.”^[^ ^17^, ^18^ ^]^ Overall, these observations, along with the fact that high mitotic rates correlate with the pluripotent state of cells, strongly suggest that a low fidelity of chromosome segregation is an inherent trait of hPSCs, explaining a relatively high incidence of aneuploidies in hPSCs.

### Short G_1_ causes DNA replication stress

Apart from the low chromosome segregation fidelity, hPSCs also exhibit heightened levels of genome damage, in the form of deleterious double‐strand breaks (DSBs), compared to their isogenic differentiated counterparts.^[^ ^20^, ^21^ ^]^ DSBs are a particular threat to genome stability, as they can give rise to a range of chromosomal rearrangements and/or loss of heterozygosity.^[^ ^22^ ^]^ High levels of genome damage in hPSCs are thought to arise, at least partly, from the rapid progression of hPSCs through the cell cycle. To enable their rapid proliferation and self‐renewal, hPSCs possess a shortened G_1_ phase compared to lineage‐committed and differentiated cells.^[^ ^23^ ^]^ The short G1 may impact the genome integrity of hPSCs as they have less time than their somatic counterparts to prepare metabolic resources, in particular nucleotides, required for the ensuing DNA replication. Consequently, the impeded DNA replication process, termed replication stress, manifests as slower rates of replication fork progression and an increased number of sites of replication initiation (origins of firing) in hPSCs compared to somatic cells.^[^ ^21^ ^]^ The finding that supplementing hPSC cultures with exogenous nucleosides alleviates replication stress, reduces DNA damage and its subsequent effects on genome instability,^[^ ^21^ ^]^ provides not only the validation that replication stress underpins genome damage in hPSCs, but also offers an important practical means to minimizing DNA damage in these cells.

### Hyper‐transcription and DNA replication stress promote chromosome mis‐segregations

Another potential cause of the genome damage and subsequent low chromosome segregation fidelity may lie in hPSCs’ hyper‐transcriptive nature. Unlike differentiated cells, but similar to cancer, pluripotent cells have been shown to exist in a state of increased transcription in order to support their rapid proliferation and cell growth.^[^ ^24^, ^25^ ^]^ This state is referred to as hyper‐active transcription, or hyper‐transcription, and could generate constitutive DNA replication stress in hPSCs.^[^ ^24^ ^]^ Mechanistically, the DNA replication stress may result from transcription‐replication conflicts, whereby hyper‐transcription introduces ongoing transcription along the DNA that hinders replication fork progression during DNA replication,^[^ ^26^, ^27^ ^]^ raising the probability of fork collapse, fork breakage, and subsequent DNA damage via DSBs.^[^ ^24^ ^]^ Extrapolating from a study performed on human pigmented epithelial cells, replication stress may also cause premature centriole disengagement in G_2_,^[^ ^28^ ^]^ an abnormality known to lead to merotelic spindle‐kinetochore attachments, and ultimately, non‐disjunctions that originate aneuploidies.^[^ ^29^ ^]^ However, more data is required to further solidify this assumption in hPSCs.

Taken together, cell autonomous traits in hPSCs, such as a short G_1_ phase, possibly in combination with hyper‐transcription, give rise to constitutive DNA replication stress. The replication stress in turn underpins hPSCs’ propensity for deleterious DSBs and their poor chromosome segregation fidelity.^[^ ^17^, ^21^, ^30^ ^]^

## LOW MUTATION RATE IN HPSCS IS MAINTAINED BY HIGH RATES OF APOPTOSIS

**FIGURE 1:**
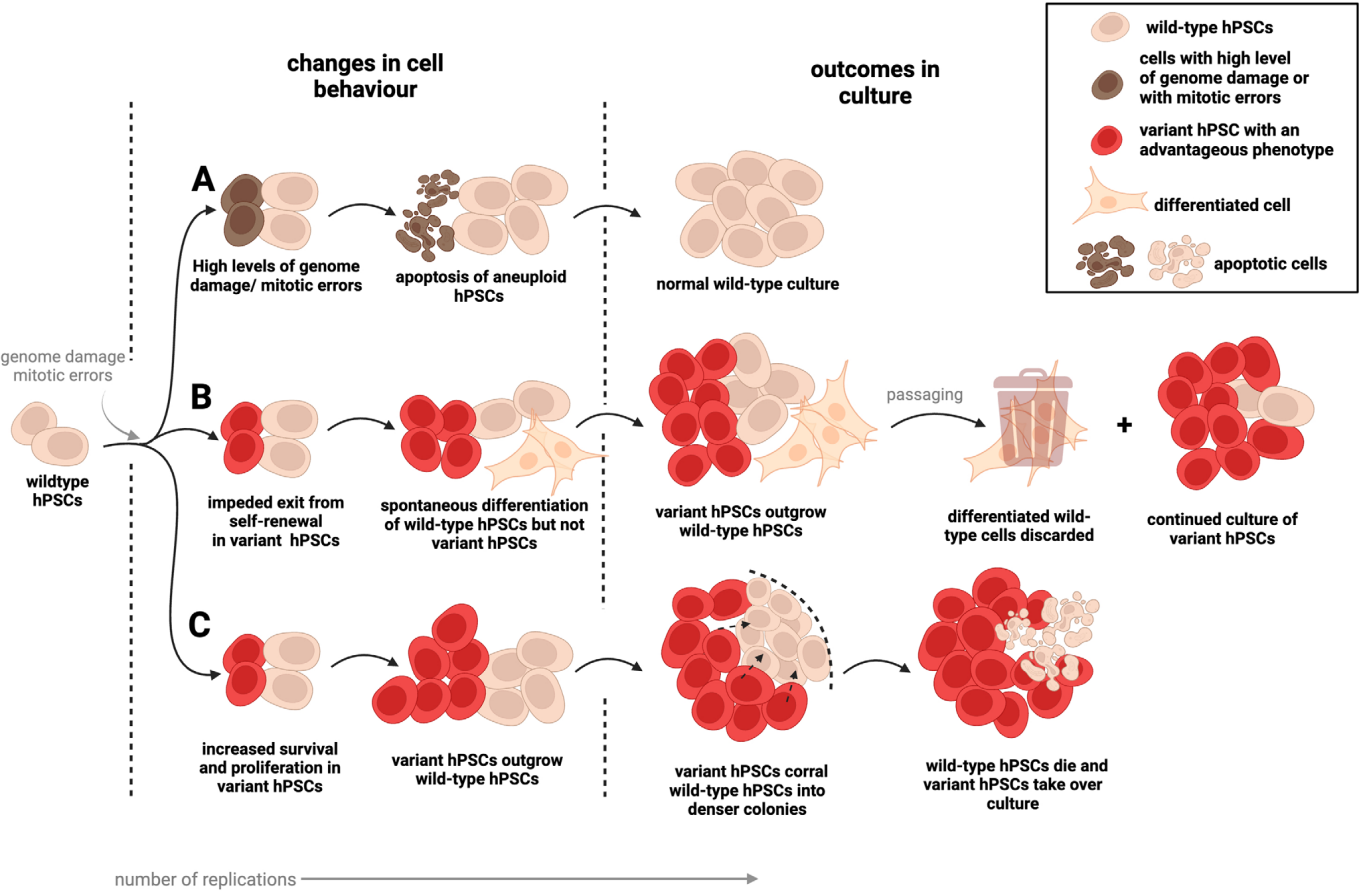
The processes of mutation and selection lead to the appearance of genetically variant cells in hPSC cultures. (A) Low mutation rate in hPSCs is maintained by the heightened susceptibility of hPSCs to apoptosis. (B) Resistance to differentiation in variant hPSCs can lead to their cultural dominance. (C) Variant hPSCs, through enhanced survival and proliferation, can outcompete hPSCs. Additionally, some variant hPSCs can selectively eliminate wild‐type counterparts, therefore rapidly dominating cultures. (Original figure created in BioRender.com).

The observation that hPSCs suffer from high levels of genome damage and mitotic errors may lead to a logical conclusion that their genomes are particularly unstable. However, the experimental evidence goes strongly against this presumption. For instance, in a large‐scale international study, more than 66% of 125 lines examined at an “early” and “late” passage remained euploid, a finding that suggests that hPSCs are generally able to retain a stable karyotype.^[^ ^10^ ^]^ Mutation rate at an SNV and small indel level was also directly assessed in a couple of independent studies, again demonstrating a relatively low overall mutation rate.^[^ ^31^, ^32^ ^]^ So, how is it possible that despite a high frequency of mitotic errors and heightened genome damage, features also characteristic of many genetically unstable cancer cells, hPSCs nonetheless retain a relatively low mutation rate? The answer lies in an increased vulnerability of hPSCs to apoptosis in response to genome damage and mitotic errors. Indeed, unlike somatic cells that tend to pause in order to repair their damaged genomes or resolve mitotic errors, hPSCs instead tend to rapidly commit to apoptosis,^[^ ^18^, ^21^, ^33^ ^]^ thereby eliminating damaged cells from their population (Figure 1A). Strikingly, high levels of apoptotic cells are also seen in human preimplantation development,^[^ ^34^ ^]^ suggesting that the hypersensitivity to apoptosis is a feature of pluripotent cells, deployed as a mechanism for suppressing the incidence of mutations in pluripotent cell populations. While seemingly effective in maintaining the low mutation rate, the reliance of pluripotent cells on apoptosis may make them susceptible to the accumulation of genetic changes conferring an anti‐apoptotic resistance.^[^ ^35^, ^36^, ^37^, ^38^ ^]^

## CULTURE SELECTION: RECURRENT GENETIC CHANGES BESTOW GROWTH ADVANTAGE

Despite the overall low mutation rate in hPSC populations,^[^ ^18^, ^31^ ^]^ the prolonged culture of a very large number of cells nonetheless creates opportunities for the selection of cells with specific advantageous traits. Indeed, in expanding hPSC populations, just like in any living ecosystem, the principles of Darwinian natural selection hold true, with hPSC variants that are “fitter” for prolonged cultures outcompeting “less fit” wild‐type hPSCs and non‐advantageous variants in culture.^[^ ^5^ ^]^ As a corollary, the repeated occurrence of particular genetic changes in hPSCs may be explained by the positive selection for those variants that confer “fitter” phenotypes to hPSCs.^[^ ^4^, ^5^, ^8^, ^9^, ^10^ ^]^ Consistent with this notion, many of the recurrent genetic changes have indeed been shown to endow hPSCs with enhanced survival, reduced apoptosis, and overall increased proliferation,^[^ ^5^, ^8^, ^35^, ^36^, ^39^ ^]^ which give variant hPSCs an advantage in culture compared to co‐existing wild‐type cells (Table 1). Additionally, variant hPSCs with resistance to differentiation could also achieve culture dominance by having a higher chance of being passaged^[^ ^40^, ^41^, ^42^ ^]^ (Figure 1B). Finally, although the selective advantage of variants is typically considered and studied in the light of cell‐autonomous effects that genetic changes confer onto variant cells, the dynamics of the variant overtake of cultures is also dictated by additional factors, such as the nature of variants’ interaction with wild‐type cells.^[^ ^43^ ^]^ Indeed, some variants were found to outcompete wild‐type cells not only through having reduced apoptotic rates and faster cell cycle but also through their ability to eliminate wild‐type cells, for example, in conditions of high cell density in which the cells are competing for space^[^ ^43^ ^]^ (Figure 1C).

**TABLE 1:** Recurrent genetic changes commonly detected in hPSCs, the altered phenotypes that they confer onto variant hPSCs, and the genes responsible for altered phenotypes.

Recurrent genetic change	Associated phenotypes	Driver gene or genes associated with variant phenotypes
1q gain	Increased survival and proliferation in feeder‐free cultures^[^ ^37^, ^38^ ^]^; culture conditions‐ dependent selective advantage,^[^ ^37^ ^]^ increased tolerance of genome damage compared to wild‐type cells^[^ ^37^, ^38^ ^]^; altered differentiation ability^[^ ^38^, ^56^ ^]^; persistence during differentiation.^[^ ^38^ ^]^	*MDM4* amplification^[^ ^37^, ^38^ ^]^
12p gain	Increased proliferation and teratocarcinoma formation^[^ ^50^ ^]^; altered differentiation ability.^[^ ^75^ ^]^	Enhanced proliferation and impaired differentiation: *NANOG* amplification.^[^ ^75^ ^]^
17q gain	Increased proliferation and reduced apoptosis compared to wild‐type hPSCs^[^ ^43^ ^]^; altered differentiation pattern toward neural lineage.^[^ ^54^ ^]^	Altered differentiation pattern: amplified WNT3 and WNT9B.^[^ ^54^ ^]^
18q loss	Decreased neuroectodermal differentiation.^[^ ^57^ ^]^	Loss of *SALL3*.^[^ ^57^ ^]^
20q gain	Increased proliferation and reduced apoptosis compared to wild‐type hPSCs,^[^ ^35^, ^36^ ^]^ increased genome instability,^[^ ^18^ ^]^ and disrupted differentiation.^[^ ^40^ ^]^	Growth advantage and impaired differentiation: *BCL2L1* amplification.^[^ ^35^, ^36^, ^40^ ^]^
Single nucleotide variants in *TP53*	Growth advantage over wild‐type hPSCs^[^ ^14^ ^]^; persistence of *TP53* mutations during differentiation^[^ ^48^ ^]^; altered differentiation patterns.^[^ ^48^ ^]^	Dominant negative mutations in *TP53*.^[^ ^14^, ^48^ ^]^

Studying the genotype‐phenotype relationship in variant hPSCs presents opportunities to uncover the molecular circuitry underlying the altered hPSC behavior and fates. The first example of a recurrent hPSC variant for which the underpinning driver gene was identified is the copy number variant (CNV) of chromosome 20q.^[^ ^35^, ^36^ ^]^ The driver gene identification in this CNV was facilitated by the relatively small common amplicon identified across multiple cell lines harboring the 20q CNV.^[^ ^10^, ^35^, ^36^ ^]^ Genetic manipulation experiments of a range of candidate genes present in the minimal amplicon demonstrated that only the overexpression of BCL2L1, or specifically, its anti‐apoptotic form BCL‐XL phenocopies 20q CNV, hence demonstrating that the amplification of *BCL2L1* drives the selective advantage of this recurrent variant.^[^ ^35^ ^]^ More recently, two independent studies highlighted *MDM4* as the likely driver gene for recurrent gains of chromosome 1q.^[^ ^37^, ^38^ ^]^ MDM4 is a negative regulator of TP53 and, like BCL2L1,^[^ ^44^ ^]^ *MDM4* is also frequently amplified in human cancers.^[^ ^45^, ^46^, ^47^ ^]^ A further parallel of BCL2L1 and MDM4 is that they both provide an anti‐apoptotic resistance to variant hPSCs,^[^ ^37^, ^38^ ^]^ raising the possibility that the high level of apoptosis represents the key selective pressure operating in hPSC populations.

Apart from the gains of chromosome 1q and 20q CNVs, the driver genes for other recurrent karyotypic aberrations remain to be determined. The increased usage of higher‐resolution techniques, such as SNP arrays, optical genome mapping, and next‐generation sequence‐based karyotyping methods, over traditional karyotyping (e.g., G‐banding) techniques, may help with further narrowing down the minimal amplicons on each of the recurrently gained/lost chromosomes. However, the absence of a narrow minimal region may suggest the requirement for two or more distally placed genes on an amplified chromosome in driving the advantageous phenotype.

Unlike in aneuploidies and CNVs in which the large number of genes typically makes the identification of driver genes complicated, SNVs occurring in the coding regions of a gene can more directly pinpoint the underlying culprit. Clearly illustrating this point is the identification of dominant negative mutations in *TP53* that, worryingly, were the same dominant negative mutations most observed in human cancers.^[^ ^14^, ^15^, ^48^ ^]^ Another gene associated with hematopoietic malignancies, *BCOR*, was also recently identified as recurrently mutated in hPSCs.^[^ ^13^ ^]^ The full extent of recurrent SNVs is yet to be revealed as traditional cytogenetic techniques make way to newer sequencing‐based technologies, capable of nucleotide‐level resolution in the detection of genetic changes.^[^ ^4^ ^]^

## IMPLICATIONS: CONSEQUENCES OF GENETIC CHANGES ON THE APPLICATIONS OF hPSCs

Changes in hPSC behavior due to genetic aberrations may present significant obstacles for the future use of these cells in research and therapy.^[^ ^3^, ^49^ ^]^ The presence of genetic aberrations in conjunction with uncontrolled proliferation of genetically abnormal cells are known hallmarks of cancer, hence this resemblance of recurrent hPSC variants with cancerous cells raises a worrying question of whether hPSC variants could share the same malignant properties as cancerous cells, and if they indeed possess neoplastic potential. This possibility was investigated in a study that compared cells with and without a recurrent karyotypic aberration, trisomy 12.^[^ ^50^ ^]^ Although this study pointed to a higher propensity of karyotypically abnormal hPSCs compared to wild‐type cells in generating malignant teratocarcinoma‐like tumors in mice,^[^ ^50^ ^]^ this association does not appear entirely straightforward. In a more recent study carried out by the International Stem Cell Initiative, the induction of malignant teratocarcinomas versus benign teratomas in mice did not correlate with the karyotype of the transplanted hPSCs, with some abnormal hPSCs giving rise to teratomas and, conversely, euploid hPSCs generating teratocarcinomas.^[^ ^51^ ^]^ Of course, undifferentiated hPSCs are not the intended target cell type for cell therapies and, additionally, any potential residual undifferentiated hPSCs in clinical samples can be nowadays identified with a rather high sensitivity.^[^ ^52^, ^53^ ^]^ Hence, rather than focusing on the potential tumorigenicity and malignancy of hPSCs, more pertinent questions are whether genetically variant cells that arise during hPSC expansion persist during differentiation and whether they could confer tumorigenic properties onto differentiated cells. The answers to these questions are still incomplete, but the persistence of variants during differentiation has been demonstrated for at least some of the recurrent abnormalities, including gains of chromosome 1q, 17q, and *TP53* mutations.^[^ ^14^, ^38^, ^48^, ^54^ ^]^ These findings confirm the need to screen the final cellular product for the presence of genetic changes and highlight the urgency of assessing their functional significance for specific intended clinical applications.

**FIGURE 2:**
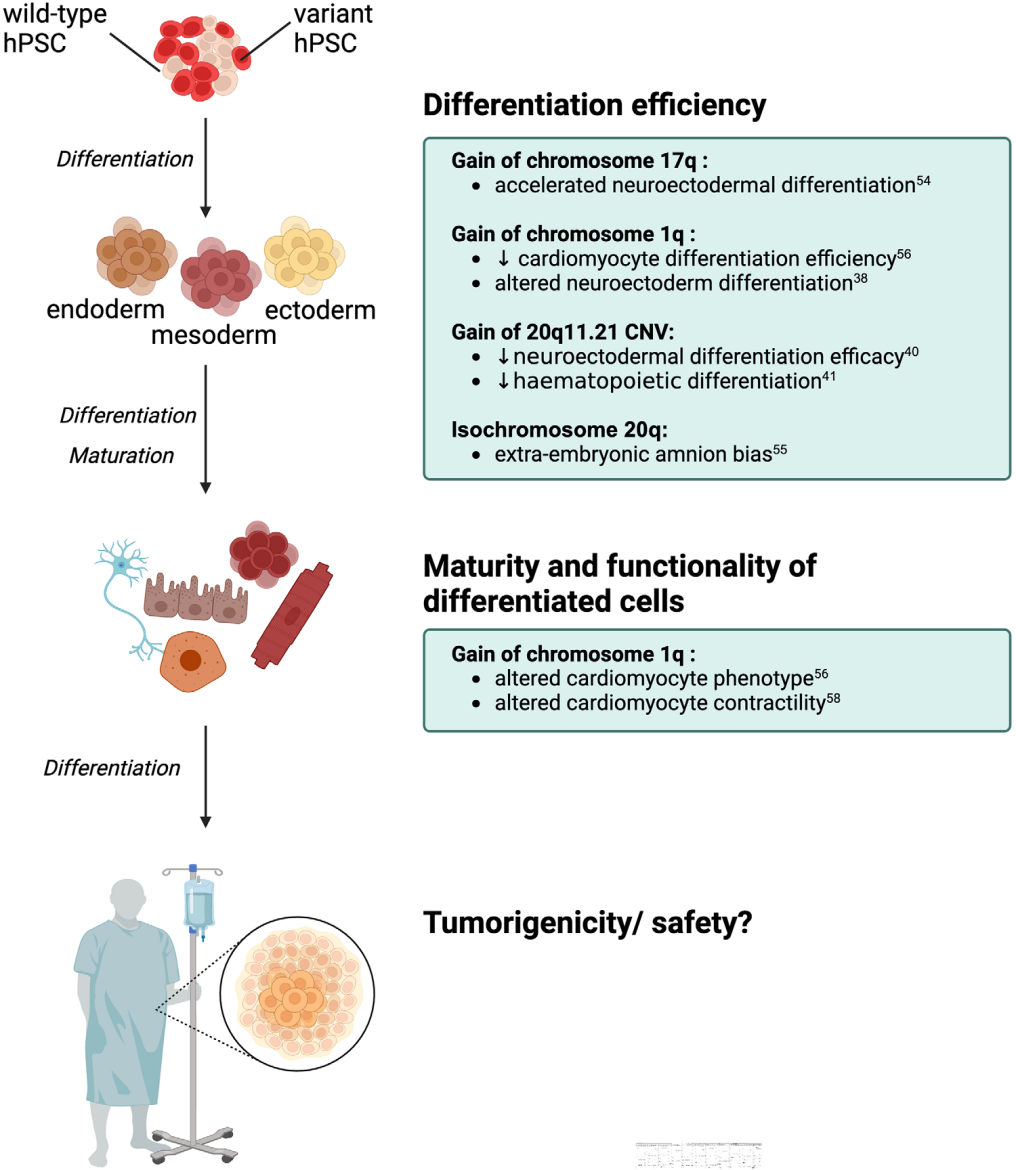
The presence of variant hPSCs in culture can impact the differentiation efficiency of cells, functionality, and maturity of differentiated cell types, and may present a safety issue in regenerative medicine. (Original figure created in BioRender.com).

While the potential tumorigenicity of variant hPSCs is at the forefront of safety concerns regarding the uses of hPSCs in regenerative medicine, the potential detrimental effects of genetically variant hPSCs do not stop there. As discussed above, recurrent hPSC variants, such as those with a chromosome 12p, 17q, 20q gain, or 18q loss, or variants with SNVs in *TP53* have also been observed to be differentiation‐defective, either failing to differentiate upon being subjected to differentiation cues or showing bias toward certain cell lineages, producing unwanted cell types^[^ ^40^, ^41^, ^48^, ^54^, ^55^ ^]^ (Table 1). For instance, in a study investigating the impact of chromosome 1q gain on cardiomyocyte differentiation, hPSC variants with a gain of chromosome 1q failed to differentiate after being exposed to established concentrations of Wnt activators that successfully induced differentiation of their isogenic wild‐type counterparts.^[^ ^56^ ^]^ Similarly, recurrent *TP53* mutations also affected the differentiation ability of cells, yielding profound differences in the transcriptional profiles of cells.^[^ ^48^ ^]^ Finally, variants with a loss of chromosome 18q exhibited a decreased capacity for neuroectodermal differentiation as a consequence of losing a copy of *SALL3* located on chromosome 18q.^[^ ^57^ ^]^ Hence, differentiation‐defective hPSC variants could generate very impure hPSC‐derived progenitors consisting of large portions of either undifferentiated cells and/or other unintended differentiated cell types, thus hampering the efficacy of stem cell‐based therapies. Further, variants arising in hPSCs may also affect the phenotype, maturity, and functionality of differentiated cell types,^[^ ^40^, ^41^, ^58^, ^59^ ^]^ which would in turn also stand to jeopardize the efficacy of cell therapies (Figure 2).

The presence of hPSC variants could also compromise studies using hPSCs for basic research, disease modeling, and drug discovery. For instance, altered responsiveness of variant hPSCs to differentiation cues may lead to unpredictable outcomes, thus negatively impacting the reproducibility of experiments. Moreover, the presence of a culture‐acquired variant in an hPSC‐based model of a genetic disease may lead to an altered behavior of cells, not reflective of the actual disease supposedly studied. Recognizing these possibilities, the International Society for Stem Cell Research has recently developed “Standards for Use of Human Stem Cells in Research,” which stipulate the need to regularly monitor hPSC cultures for the presence of genetic changes to help mitigate these risks.^[^ ^60^ ^]^ Diminishing costs and increased accessibility of technologies, such as next‐generation sequencing^[^ ^61^ ^]^ and optical genome mapping,^[^ ^62^ ^]^ are expected to facilitate the regular high‐resolution monitoring of hPSC genomes (reviewed in Andrews et al.^[^ ^3^ ^]^).

## STRATEGIES FOR SUPPRESSING GENETIC VARIANTS

Currently, there remains a need to develop efficient strategies to minimize variant occurrence during hPSC culture. Studies focused on the mechanistic understanding of the processes of mutation and selection in hPSC cultures are paving the way for progress in this area. For instance, the addition of nucleosides to the hPSC medium,^[^ ^21^ ^]^ growing hPSCs under conditions of low oxygen tension,^[^ ^31^, ^32^ ^]^ and reducing the opportunities for genome damage‐inducing media acidification^[^ ^63^ ^]^ all represent promising strategies for minimizing the levels of genome damage and mitotic errors that ultimately predispose hPSCs to the acquisition of genetic changes.

In addition to a further reduction of mutation rates, there is also a lot of scopes to devise strategies to reduce the selective advantage of variants and therefore suppress their dominance in hPSC cultures. A thorough understanding of culture conditions and parameters that are selected for particular variants is pivotal for achieving these goals. Yet, identifying the association of variants with culture conditions is not trivial, as it requires longitudinal studies of a very large number of samples maintained under different regimens. Recently, we took a different approach to addressing this issue. Utilizing the long temporal database of hPSC karyotypes collated by WiCell through routine monitoring of hPSC samples submitted by diverse groups, we explored trends in karyotypic aberrations with respect to culture conditions.^[^ ^37^ ^]^ The rich dataset allowed us to make an association, which we subsequently empirically tested. Specifically, we demonstrated a culture condition‐dependent advantage of variants with a gain of chromosome 1q, which outcompete wild‐type counterparts in feeder‐free (E8/vitronectin), but not feeder‐based conditions.^[^ ^37^ ^]^ This context‐dependent behavior of chromosome 1q variants explains the rise in the prevalence of 1q gains in hPSC karyotypes in recent years while providing an opportunity to pinpoint the specific selective pressure that the variants are adapted to.^[^ ^37^ ^]^ Additionally, this study provided a paradigm for how establishing and maintaining a curated database of hPSC genetic data and associated metadata (such as culture conditions) could be used for identifying trends in variant appearance through further retrospective analyses in the future.

Finally, it would be advantageous to have at our disposal strategies that permit the selective elimination of genetically variant cells once they are detected in expanding cultures. Along these lines, statins were shown to eliminate variant hPSC with amplification of chromosomes 12 and 17,^[^ ^64^ ^]^ while BH3 mimetics were effective in eliminating genetically variant hPSCs with a gain of 20q11.21 CNV.^[^ ^65^ ^]^ Nonetheless, these approaches were tested only on a very limited number of variants, and hence their usefulness in eliminating a range of variants and mechanisms of action still needs to be confirmed. Gaining an in‐depth understanding of the molecular underpinnings of variant behavior may provide further, more targeted opportunities to eliminate variant cells from culture.

## FUTURE PERSPECTIVES ON ELUCIDATING GENETIC VARIANTS: WHICH VARIANTS SHOULD WE CARE ABOUT?

Notwithstanding, the need to minimize the appearance of variant hPSCs, future efforts should also be directed toward the understanding of *which* genetic variants should be considered “deleterious,” as some variants may not pose as serious an issue in certain contexts. For example, a recent study demonstrated that variant hPSCs with an isochromosome 20q (iso20q) committed to apoptosis when subjected to spontaneous retinal pigmented epithelium (RPE) differentiation via the withdrawal of FGF2.^[^ ^55^ ^]^ Therefore, iso20q variants may not be a cause for worry in the context of producing RPE cells for the treatment of age‐related macular degeneration, as iso20q variants would be eliminated and selected against during the differentiation process. However, unless such elimination during differentiation is proven to be 100% effective, it may not be acceptable for applications in cell therapy. Moreover, it remains to be seen whether iso20q variants would persist during differentiation to another target cell type. Finally, although most research efforts thus far have been rightfully allocated to the study of recurrent genetic changes in hPSCs, it would also be crucial to elucidate non‐recurring variants that may present in particular lines or differentiated cell preparations.^[^ ^66^ ^]^ Taken together, procuring novel approaches for monitoring, prevention, and active elimination of variants are needed to provide effective ways forward for maintaining wild‐type hPSCs for downstream applications in research and therapy.

Apart from the genetic alterations in the nuclear DNA, mutations in mitochondrial DNA (mtDNA) have also been identified in hPSCs.^[^ ^11^, ^67^, ^68^, ^69^ ^]^ In contrast to early‐passage hESCs, which have a relatively low mtDNA variant load, hiPSCs, and late‐passage hESCs display a much higher variant load.^[^ ^70^ ^]^ Variants in mtDNA were shown to persist during hPSC differentiation and cause metabolic dysfunction of cells.^[^ ^69^ ^]^ These observations suggest that in addition to monitoring nuclear DNA, the regular monitoring of mtDNA is also warranted during hPSC expansion in culture.^[^ ^71^ ^]^ Thus, future efforts in the field should identify alterations in hPSC phenotype brought about by mtDNA mutations and determine whether and to what extent such mutations affect downstream applications of hPSCs in research and cell therapy.

The challenge of discerning the functional consequences of variants seems daunting, given the possible spectrum of variants and the variety of different contexts in which they would need to be tested. A progress on this front will undoubtedly depend on a range of approaches that encompass, for example, a careful curation of variants and their known biological consequences, alongside functional assays that can read out variant effects. Finally, the advent of artificial intelligence technologies may offer unprecedented opportunities to integrate multiple datasets and expedite the process of variant interpretation.^[^ ^72^ ^]^

## CONCLUSION

Twenty years on from the initial report of recurrent genetic changes in hPSCs, the map of recurrent abnormalities has become well established, alongside significant albeit still incomplete inroads into the molecular insights of their causes and consequences. However, pertinent questions remain as to how to predict the functional consequences of every detected variant. The issue of interpretation of genetic variants is not unique to the field of hPSCs and cell therapy. In fact, the so‐called variants of unknown significance pose a dilemma, for example, in the diagnosis of genetic disorders and cancer.^[^ ^73^, ^74^ ^]^ Given the commonality of this issue from cell therapy to cancer, perhaps a sensible way forward may be to join forces with other fields in order to catalogue and decipher human genome variation. In that regard, while overall undesirable for cell therapy, recurrent genetically variant hPSCs will provide an exceptional and potent tool for studying the effect of genome variation on human cell fate and function.

## AUTHOR CONTRIBUTIONS

J.P.V. and I.B. wrote the manuscript.

## CONFLICT OF INTEREST STATEMENT

Ivana Barbaric is a member of the scientific advisory board of WiCell.

## Data Availability

Data sharing is not applicable to this article as no new data were created or analyzed in this study.
